# Tumor necrosis factor superfamily ligand mRNA expression profiles differ between humans and mice during homeostasis and between various murine kidney injuries

**DOI:** 10.1186/s12929-017-0383-3

**Published:** 2017-09-19

**Authors:** Satish Kumar Devarapu, Julia Felicitas Grill, Junhui Xie, Marc Weidenbusch, Mohsen Honarpisheh, Volker Vielhauer, Hans-Joachim Anders, Shrikant R. Mulay

**Affiliations:** 1 Medizinische Klinik und Poliklinik IV, Klinikum der Universität München, Munich, Germany; 2 Department of Endocrinology, Tongji Hospital, Tongji Medical College, Huazhong, University of Science and Technology, Wuhan, China; 3 Nephrologisches Zentrum, Medizinische Klinik und Poliklinik IV, Klinikum der Universität München, Schillerstr. 42, D-80336 Munich, Germany

**Keywords:** Crystal nephropathy, Ischemia reperfusion injury, Chronic kidney injury, Anti-GBM, Tumor necrosis factor, Tumor necrosis factor ligands, TNF superfamily

## Abstract

**Background:**

Several tumour necrosis factor (TNF) based therapeutics have already been approved for human use and several others are emerging. Therefore, we determined the mRNA expression levels of the TNF superfamily ligands (TNFSF) – e.g. TNF-α, lymphotoxin (LT)-α, LT-β, Fas-L (CD95-L), TNF-related apoptosis-inducing ligand (TRAIL), TNF-related weak inducer of apoptosis (TWEAK), 4-1BBL, OX40-L (CD252) and amyloid precursor protein (APP) in healthy human and mouse solid organs.

**Methods:**

We used quantitative real time-PCR to analyse mRNA expression levels of TNFSF ligands. Murine models of acute ischemic renal injury, chronic oxalate nephropathy, and immune complex glomerulonephritis were used. Renal injury was assessed by PAS staining, and infiltrating immune cells were analysed by immunohistochemistry. Data was analysed using non-parametric ANOVA (non-parametric; Kruskal-Wallis test).

**Results:**

We observed significant differences in the mRNA expression levels of TNFSF ligands in human and mouse solid organs. Furthermore, we determined their mRNA expressions during acute and chronic kidney injuries in mice. Our data demonstrate that the mRNA expression levels of TNFSF vary depending on the type of tissue injury – for example, acute ischemic renal injury, chronic crystalline nephropathy, and immune complex glomerulonephritis. In addition, we observed that mRNA expressions of TNFSF ligands are differentially regulated during the course of a transient ischemic renal injury (IRI) and chronic kidney modelling. We observed that TNF-α, LT-β, and 4-1BBL were significantly upregulated during the progression of IRI and crystal-induced chronic kidney disease (CKD), whereas only 4-1BBL and TNF-α were significantly upregulated and LT-β was significantly downregulated during the progression of immune complex glomerulonephritis. The mRNA expression of Fas-L was higher during IRI whereas it decreased in a time dependent manner during the progression of crystal-induced CKD.

**Conclusion:**

We conclude that the injury- and species-specific differences of TNFSF ligands must be considered in order to avoid the misinterpretation and wrong conclusions during data extrapolation between species.

## Background

The potential of tumor necrosis factor (TNF) as a therapeutic target was exploited and has been well characterized in various diseases soon after its discovery in 1975 [1, 2]. Until today, research in this field unveiled the existence of 19 TNF superfamily (TNFSF) proteins that signal through 29 receptors [3]. The TNFSF members are expressed widely and play major roles in immune responses, inflammation, cell homeostasis, and tissue repair [4, 5]. In addition, they also contribute to disease pathogenesis, and therefore, are also referred as “double-edged swords” [3]. Most of the TNFSF members are type II transmembrane proteins, while some can be secreted proteins with biological activity [6, 7]. Originally, macrophages were reported as a source of TNF [1]. However, later on, a cytokine lymphotoxin (LT), which has similar cytotoxic effects like TNF, was reported to be secreted by B lymphocytes [8]. In 1984, the homology between TNF and LT was unveiled when they were purified to homogeneity and their amino acid sequences were determined [9, 10]. This initiated the hunt for more TNF-like proteins leading to the discovery of 19 TNFSF ligands until today [3].

The TNFSF ligands have C-terminal TNF homolog domain facilitating self-trimerization and receptor binding. The TNF-receptor superfamily has two domains consisting of an extracellular domain for ligand binding and a cytoplasmic tail domain with adaptor proteins for signal cascade activation [6, 7]. After binding to the respective receptors, all members of the TNFSF activate pathways that involve NF-ĸB, JUN N-terminal kinase (JNK), p42/44 mitogen-activated protein kinase (MAPK), p38 MAPK and generation of reactive oxygen species (ROS) that orchestrate the pleiotropic effects of TNFSF ligands [11].

A huge amount of research on TNFSF ligands is conducted in mice and the observations are often extrapolated to human conditions. However, several discrepancies in organ-specific expression levels of pattern recognition receptors (PRRs), C-type lectin receptors (CLRs), TLR accessory molecules as well as regulated necrosis-related molecules, between species has already been reported [12–18]. Therefore, we hypothesized that similar organ- and species-specific differences might exist in TNFSF ligands expressions, and hence, determined their mRNA expression profiles in human and mice organs. Furthermore, we checked their expression profiles in murine acute tissue injury and chronic (progressive and immune complex-mediated) tissue remodeling.

## Methods

### Human solid organ cDNA preparation

Total RNA from healthy human solid organ were purchased from Clontech, Mountain View, CA. From each individual sample, an equal amount of total RNA was used as a template in cDNA preparation using Superscript II (Invitrogen). As only a single pool was available for each organ, no studies on biological replicates allowing statistics could be performed. However, we used technical triplicates of each pooled sample. Usage of human solid organ RNA samples obeys the purchase and import of Clontech local laws and regulations.

### Mouse solid organ cDNA preparation for qRT-PCR experiments

Ten-twelve weeks old, adult C57BL/6 N male mice were purchased from Charles River, Sulzfeld, Germany. Group of five mice in a cage were housed in specific pathogen-free conditions with ad libitum food and water. Mice were sacrificed under general anesthesia by cervical dislocation. From freshly collected tissues, RNA was isolated as described [16]. Briefly, all organs immediately after harvest were placed in RNA later solution and RNA was isolated with an equal amount of tissue mass using Pure Link RNA Mini Kit (Ambion, Germany) according to the manufacture instructions. All RNA samples were subjected to DNAse enzyme treatment and additional washing steps were performed to remove traces of DNAse. A NanoDrop 1000 Spectrophotometer was used to estimate the RNA concentrations, only samples with absorbance 260/280 between 1.95 and 2.05 were considered as pure RNA. The integrity of the total RNA was determined by electrophoresis on 2% (*w*/*v*) agarose gels as described. Further, cDNA was transcribed using 1 μg of RNA as described before [16]. Briefly, 1 μg RNA was mixed with cDNA master mix (including DTT, dNTPS, Rnasin, Acrylamide, Hexanucleotide and Superscript II), and incubated for 1 h 30 min at 42 °C. Supercript was inactivated by incubating at 90 °C for 5 min and cDNA was stored at −20 °C until further use.

### Animal studies

Renal Ischemic injury model: Groups of seven to eight week old C57BL/6 mice (*n* = 5–10) under anesthesia underwent unilateral renal pedicle clamping for 35 min followed by reperfusion for 24 h, 5 days and 10 days as a model of ischemia-reperfusion as described [19–21]. Throughout the procedure, body temperatures were maintained at 37 °C using a heating plate (Kleintier-OP-Tisch M12511, Medax GmbH, Germany) and an egg breeding device (Octagon 20 Advance, Brinsea products Ltd., UK). Injured and contralateral kidneys were harvested for RNA isolation and histology analysis. Contralateral kidneys served as internal control kidneys. Chronic oxalate nephropathy model was developed by feeding mice with an oxalate-rich diet that was prepared by adding 50 μmol/g sodium oxalate to a calcium-free standard diet (Ssniff, Soest, Germany) as previously described [22, 23]. Mice were sacrificed at day 7, 14 and 21 after exposure to oxalate-rich diet. Autologous anti-GBM glomerulonephritis model was developed by immunizing mice subcutaneously in both flanks with 20 μg rabbit IgG (Jackson ImmunoResearch Laboratories, West Grove, PA) in Freund’s complete adjuvant (Sigma-Aldrich, Deisenhofen, Germany). Three days later, mice received 150 μl nephrotoxic rabbit serum intravenously [24]. Animals were sacrificed on day 14, 21 and 42 days after anti-GBM serum challenge. Kidney samples were harvested for RNA isolation and histology analysis. All experimental procedures on animals were approved by the Regierung von Oberbayern, München, Germany, and were performed in accordance with their guidelines and regulations.

### Quantitative real time-PCR

An equal amount of RNA (1 μg) was used to prepare cDNA [17]. Complementary DNA was performed with Superscript II reverse transcriptase (ThermoFisher, Germany), 5× first-strand buffer (Thermo Fisher Germany), DTT (Invitrogen, Germany), dNTPs (GE Healthcare, Germany), linear acrylamide (Ambion, Germany), hexanucleotide (Roche, Germany) and RNasin (Promega, Germany). Reverse transcriptase reaction was performed for 90 min at 42 °C then the reaction was heated at 85 °C for 5 min using a Mastercycler pro (Eppendorf, Germany). RN-related molecule mRNA expression in human and mouse solid organs, as well as diseases model, were quantified by RT-PCR using GAPDH as housekeeper gene for human samples and 18 s for mouse samples as described previously [16]. Each PCR reaction (20 μl) involved 10 × *Taq* Polymerase Buffer, *Taq* Polymerase, dNTPs, BSA, PCR Optimizer, SYBR green dye, MgCl_2_, gene specific primers and 0.2 μl of synthesized cDNA. SYBR Green Dye fast-two step detection protocol from Light Cycler 480 (Roche, Mannheim, Germany) running program was used for amplification. Each amplification step included initiation step 95 °C, annealing step 60 °C and amplification step 72 °C and was repeated 40 times. Gene-specific primers (300 nM, Metabion, Martinsried, Germany) were used as listed in Table 1. DdH_2_O was used as negative control for target and housekeeper genes. A specific primer for each target was designed using the primer designing tool (NCBI) and In silico specificity screen (BLAST) was performed. The lengths of amplicons were between 90 and 120 bp. The kinetics of the PCR amplification (primer efficiency) was calculated for each set of primers. The efficiency-corrected quantification was performed automatically by the Light Cycler 480 based on extern standard curves describing the PCR efficiencies of the target and the reference gene [ratio = E_target_^ΔCPtarget (control − sample)^/E_ref_^ΔCPref (control − sample)^]. The high confidence algorithm was used to reduce the risk of the false positive crossing point. All the samples which rise above the background fluorescence (crossing point Cp or quantification cycle Cq) between 5 and 40 cycles during the amplification reaction were considered as detectable. The melting curves were analyzed for every sample to detect unspecific products and primer dimers. To visualize the similarity and differences in gene expression profiles among the samples, hierarchical cluster analysis were performed using algorithms incorporated in the open-source software MultiExperiment Viewer (MeV) version 4.9 [25]. Differentially expressed mRNAs were screened by Volcano Plot between log(2)(fold change) gene expression [unstandardized signal] against -log(10)(*p*-value) from the t-test [noise-adjusted/standardized signal] [26].

**Table 1:** Primers used for RT-PCR

Heading	Forward Sequence (5´➔3′)	Reverse Sequence (3´➔ 5′)	Accession Nr.	Efficiency
Human				
TNF-α	ATGGGCTACAGGCTTGTCACTC	CTCTTCTGCCTGCTGCACTTTG	NM_ 000594	1.80
LT-α	TCCGTGTTTGCTCTCCAGAGCA	ACACCTTCAGCTGCCCAGACTG	NM_ 000595	1.8
LT-β	CGTCAGAAACGCCTGTTCCTTC	GGTTTCAGAAGCTGCCAGAGGA	NM_ 000595	1.97
Fas-L	CTGTGTGCATCTGGCTGGTAGA	GGTTCTGGTTGCCTTGGTAGGA	NM_000639	1.98
TRAIL	AGCTGCTACTCTCTGAGGACCT	TGGCAACTCCGTCAGCTCGTTA	NM_003810	1.73
TWEAK	TGGAGCTGTTGATTCTGGCTTCC	AAAACACGGGCTCGAAGAGCGA	NM_003809	2.14
4-1BBL	TGGAAATCGGCAGCTACAGCCA	TCTTCCTCACGCTCCGTTTCTC	NM_003811	2.08
OX40-L	TGATGACTGAGTTGTTCTGCACC	CCTACATCTGCCTGCACTTCTC	NM_003326	2.10
APP	CCTTCTCGTTCCTGACAAGTGC	GGCAGCAACATGCCGTAGTCAT	NM_000484	2.15
Mouse				
TNF-α	GGTGCCTATGTCTCAGCCTCTT	GCCATAGAACTGATGAGAGGGAG	NM_013693	2.20
LT-α	AGCCCATCCACTCCCTCAGAAG	TGCTCTCCAGAGCAGTGAGTTC	NM_010735	1.94
LT-β	CCTGTTGTTGGCAGTGCCTATC	GACGGTTTGCTGTCATCCAGTC	NM_008518	2.22
Fas-L	GCCACACTCCTCGGCTCTTTTT	GAAGGAACTGGCAGAACTCCGT	NM_010177	2.12
TRAIL	TGGAGTCCCAGAAATCCTCA	TCACCAACGAGATGAAGCAG	NM_009425	1.95
TWEAK	ACACCGTTCACCAGCAAGTCCA	CTGCGCTACGACCGCCAGATT	NM_013749	1.93
4-1BBL	GCGTTGTGGGTAGAGGAGCAAA	CCAAGTACCTTCTCCAGCATAGG	NM_009404	2.06
OX40-L	CTGGTAACTGCTCCTCTGAGTC	GGAAGAAGACGCTAAGGCTGGT	NM_009452	1.81
APP	GCCAAGACATCGTCGGAGTAGT	TCCGTGTGATCTACGAGCGCAT	NM_177310	2.11

### Histopathology

After sacrifice, portions of harvested kidney tissues were fixed in 4% neutral-buffered formalin, followed by dehydration in graded alcohols and embedded in paraffin. For further analysis such as periodic acid-Schiff (PAS) staining or immunostaining 4 μm sections were deparaffinized, rehydrated, transferred into citrate buffer, and either autoclaved or microwave treated for antigen retrieval and processed as described [12]. For immunostaining following primary antibodies were used: Collagen1α1 (Dako, Hamburg, Germany), anti-mouse CD3, anti-mouse F4/80 (both Serotec, Oxford, UK).

### Statistics

Data were represented as mean ± standard error of the mean (SEM). Comparisons between groups were performed using non-parametric ANOVA (non-parametric; Kruskal-Wallis test). A *p*-value less than 0.05 was chosen as statistical significance.

## Results

### TNF superfamily ligands mRNA expressions in adult human tissues

**Fig. 1:**
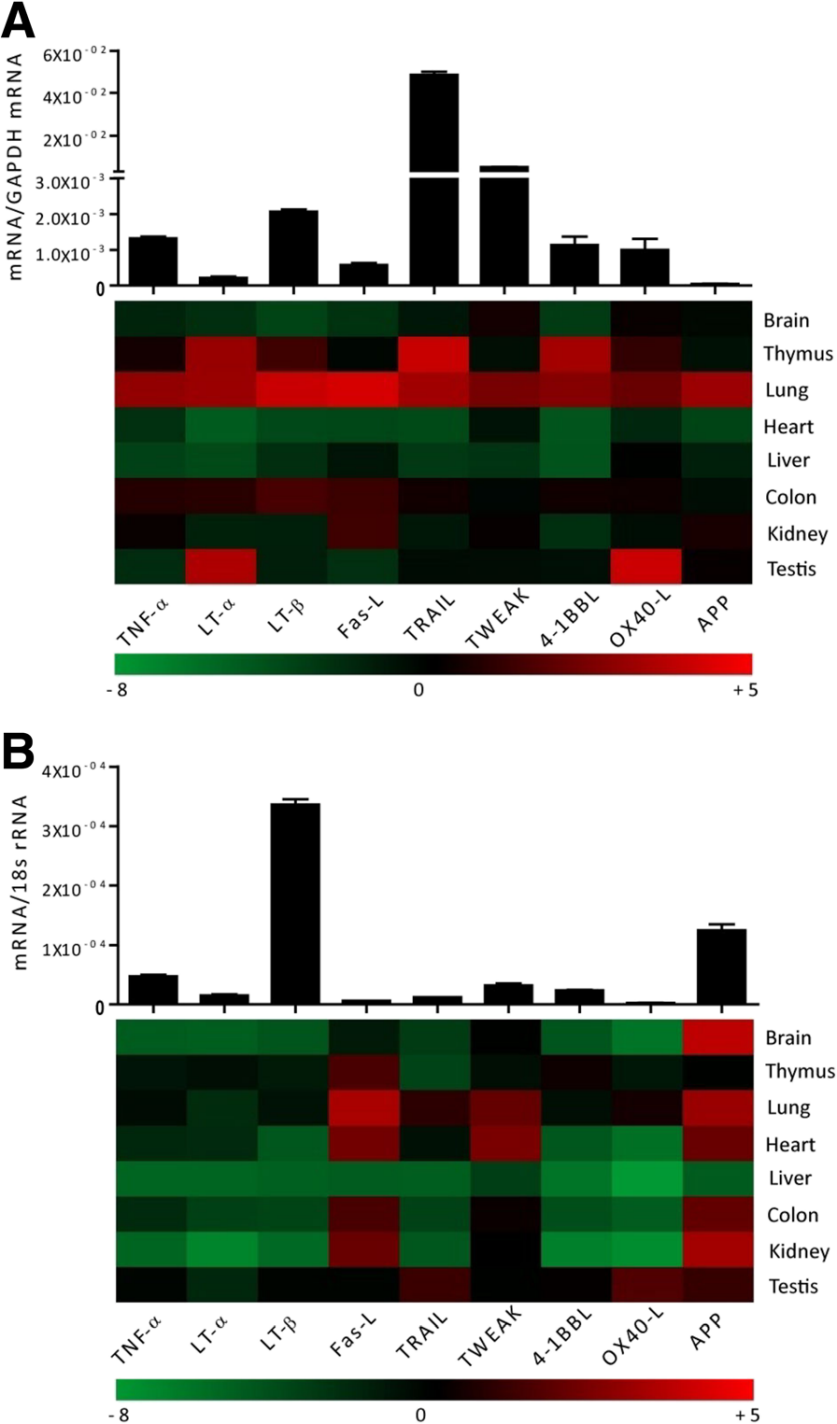
TNFSF ligands mRNA expressions in adult human and murine tissues **a** Quantitative real-time PCR analysis was performed on cDNAs prepared from pools of healthy human tissue as described in the methods and mRNA expression levels of all the organs were normalised to GAPDH mRNA expression level and spleen mRNA levels were illustrated in the form histograms. Whereas the mRNA expression levels of other organs were normalized to spleen mRNA expression levels and were represented in the heatmap. **b** cDNAs derived from five adult 12-week old C57BL/6 mice as described in methods and mRNA expression levels of all the organs were normalised to 18 s rRNA expression level and the spleen mRNA levels were illustrated in the form of histograms. Whereas the mRNA expression levels of respected other organs were normalized to spleen expression levels and were represented in the heatmap. Data represent means ± SEM

We quantified the mRNA expression levels of TNFSF ligands e.g. TNF-α, LT-α, LT-β, Fas-L (CD95-L), TNF-related apoptosis-inducing ligand (TRAIL), TNF-related weak inducer of apoptosis (TWEAK), 4-1BBL, OX40-L (CD252) and amyloid precursor protein (APP) using qRT-PCR in healthy human organs. Human spleen constitutively expressed all of these ligands with higher expression of TRAIL compared to other ligands (Fig. 1a). However, the expression of LT-α, Fas-L, and AAP remained low (Fig. 1a). In general, all of these ligands were expressed at higher levels in lungs and lower levels in brain, heart, and liver. OX40L and LT-α were expressed at higher levels in Testis. Thymus showed very high mRNA expression levels of LT-α, TRAIL, and 4-1BBL whereas those of LT-β and OX40-L were only modestly increased. All these ligand expressions in kidney were similar to spleen except for Fas-L, which was modestly high in the kidney and 4-1BBL, which was lower. The mRNA expression levels of most of the other molecules were lower in the solid organs compared to spleen. Thus, mRNA expressions of TNFSF ligands are variable in healthy human solid organs compared to spleen.

### TNF superfamily ligands mRNA expressions in adult murine tissues

**Fig. 2:**
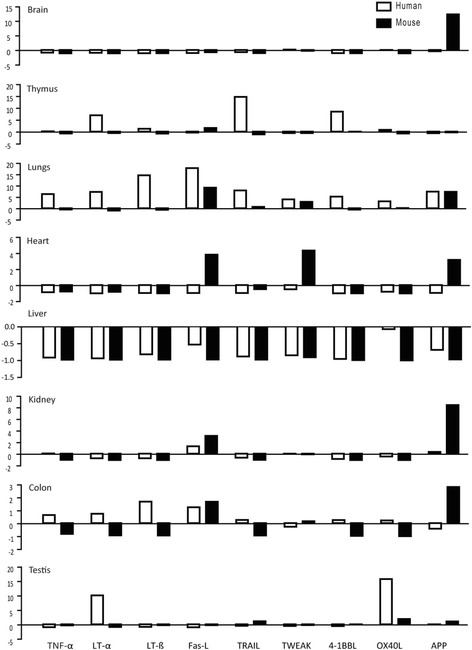
Comparison of expression profiles of TNFSF ligands in human and mice. The respective relative human (white bars) and murine (black bars) and TNFSF ligands mRNA expression levels from Fig. 1 are illustrated. The x-axis shows mRNA expression levels of the organs normalized to spleen mRNA expression levels of the respective species. The y-axis marks the fold-change in each direction. Note that the scale of the y-axis is different for each organ. Data represent means ± SEM

Next, we quantified the mRNA expression of the above-mentioned TNFSF ligands in the same organs from healthy, 10-12 weeks old, male C57BL/6 N mice. We found that mouse spleen constitutively expressed all of these ligands. However, the mRNA expression levels of LT-β and AAP were higher whereas those of Fas-L, TRAIL, and OX40L were lower in the mouse spleen (Fig. 1b). In sharp contrast to human organs, all mouse organs, except thymus, expressed higher levels of AAP (Fig. 1b). In addition, mouse spleen expressed very low mRNA expression levels of TNFSF ligands compared to human spleen. However, like human liver, mouse liver also expressed very low levels of TNFSF ligands. The mRNA expression of Fas-L was higher in thymus, lung, heart, colon, and kidney; lower in the liver, and remained unchanged in brain and testis compared to spleen. Lung also expressed higher levels of TWEAK. TRAIL was expressed at modestly high levels in lung and testis. The latter also showed modestly higher levels of OX40-L. The mRNA expression of other TNFSF ligands was markedly lower in the mouse solid organs compared to the spleen (Fig. 1b). Furthermore, we also compared the organ-specific mRNA expression of TNFSF ligands between humans and mice and observed obvious differences between them (Fig. 2). For example, mouse brain expressed very high mRNA levels of AAP (about 12 fold) compared to human brain. AAP was also expressed at higher levels in mouse kidney and lungs compared to the human kidney and lungs, respectively. TRAIL, 4-1BBL, and LT-α were expressed at higher levels in human thymus whereas OX40-L and LT-α were expressed at higher levels in human testis compared to mouse testis. Mouse heart expressed higher levels of Fas-L, TWEAK, and AAP than the human heart. Thus, humans and mice express different relative mRNA levels of TNFSF ligands.

### TNF superfamily ligands mRNA expressions in murine acute kidney injury

**Fig. 3:**
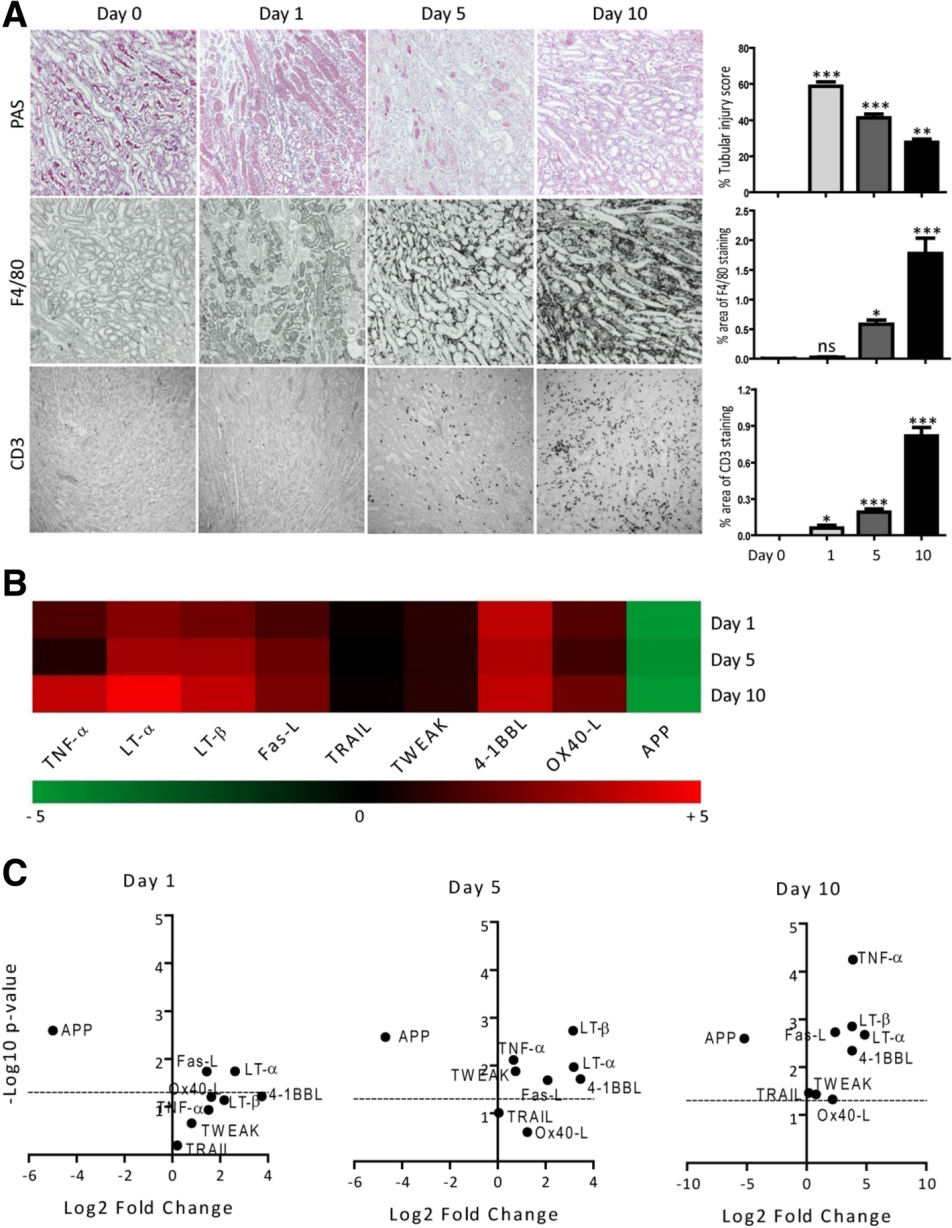
TNFSF ligands mRNA expressions in murine ischemic renal injury. IRI was induced in C57Bl6 mice as described in methods. **a** Representative images of renal sections stained with PAS or for F4/80 and CD3. Original magnification: ×100. The tubular injury, as well as F4/80 and CD3 positive area per hpf, were quantified. Data represents mean ± s.e.m. *p < 0.05, **p < 0.01, *p < 0.001. **b** Quantitative RTPCR was performed on cDNAs derived from the injured kidneys. The mRNA expression levels were normalised to 18 s rRNA expression level. The heatmap represents the relative expression of mRNA levels at day 1, 5 and 10 versus control. **c** The statistical analysis of TNFSF ligands mRNA expression levels in the injured kidneys at day 1, 5 and 10 versus control kidneys is represented using volcano graphs. *P* < 0.05 is considered statistically significant. Dotted line represent *p* = 0.05

We further studied the changes in the mRNA expression of TNFSF ligands during transient injury using a murine model of ischemic renal injury (IRI). The IRI model is characterized by acute tubular necrosis and inflammation in an injury phase (day 1), which is followed by tubular regeneration in a healing phase (day 5 and 10) (Fig. 3a) [19–21]. We observed an increase in the infiltration of F4/80+ macrophages and CD3+ T cells in the injured kidneys, which is associated with increased renal mRNA expression levels of TNFSF ligands at day 5 and 10 (Fig. 3a). The mRNA expression levels of TRAIL, TWEAK, as well as OX-40 L, remained unchanged at day 1, 5 and 10 (Fig. 3b-c). The mRNA expression levels of APP were significantly downregulated whereas that of Fas-L and LT-α was significantly upregulated at all time points (Fig. 3b-c). Moreover, the mRNA expression levels of TNF-α, LT-β, and 4-1BBL were increased only in the healing phase, at day 5 and 10 (Fig. 3b-c). Therefore, we conclude that mRNA expressions of TNFSF ligands are differentially regulated during the course of a transient injury – for example, ischemic renal injury.

### TNF superfamily ligands mRNA expressions in murine chronic kidney disease

**Fig. 4:**
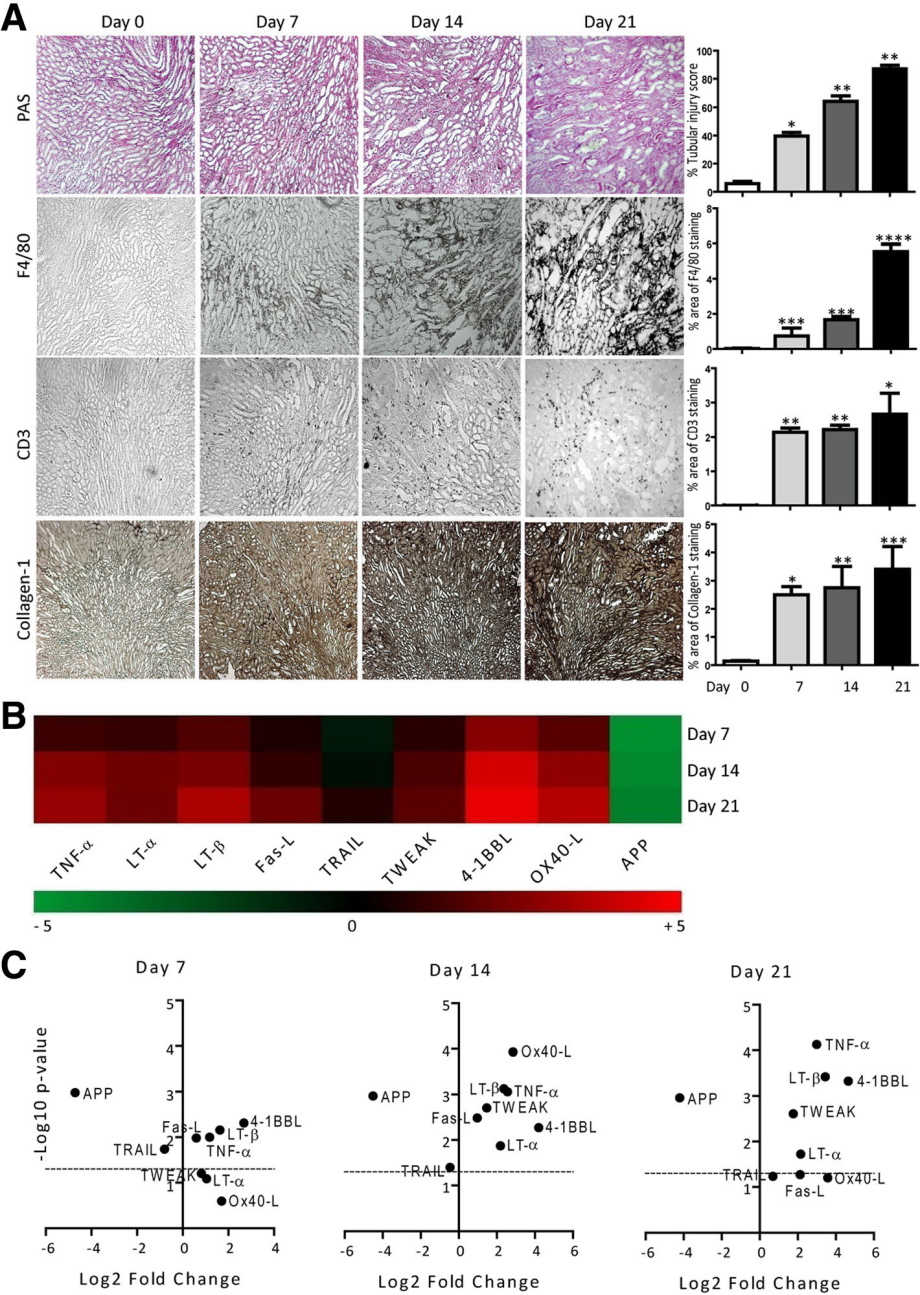
TNFSF ligands mRNA expressions in murine oxalate-induced CKD. Oxalate-induced CKD was induced in mice as described in methods. **a** Representative images of renal sections stained with PAS or for F4/80, CD3 and collagen1. Original magnification: ×100. The tubular injury, F4/80, CD3 positive cells, and collagen-1 were quantified. Data represents mean ± s.e.m. *p < 0.05, **p < 0.01, *p < 0.001. **b** Quantitative RTPCR was performed on cDNAs derived from the kidneys of mice on day 7, 14 and 21. The mRNA expression levels were normalised to 18 s rRNA expression level. The heatmap represents the relative expression of TNFSF ligands mRNA levels at day 7, 14 and 21versus day 0. **c** The statistical analysis of TNFSF ligands mRNA expression levels in the kidneys at day 7, 14 and 21 versus day 0 is represented using volcano graphs. *P* < 0.05 is considered statistically significant. Dotted line represent *p* = 0.05

We observed higher levels of mRNA expressions of TNFSF ligands in healing phase after a transient renal injury. Therefore, we next studied the changes in their expression during chronic tissue remodeling. We induced chronic kidney disease (CKD) in mice by feeding them an oxalate-rich diet for 21 days [22, 23]. Oxalate-induced CKD was associated with a progressive increase of F4/80+ macrophages and CD3+ T cells infiltrating the renal interstitial compartment as well as interstitial fibrosis from day 7 to 21 (Fig. 4a). Similar to IRI, the mRNA expression of APP was significantly downregulated at all time points. At day 7, mRNA expressions of TWEAK, LT-α, and OX-40 L remained low. The later was only induced significantly at day 14 whereas LT-α was significantly high at day 14 and 21. There were no significant differences observed in the mRNA expression of TRAIL in oxalate-induced CKD. However, mRNA expression levels of 4-1BBL, TNF-α, and LT-β were significantly higher at day 7, 14, and 21. In contrast, the mRNA expression of Fas-L significantly decreased in a time-dependent manner during the progression of oxalate-induced CKD (Fig. 4b-c). Thus, the mRNA expressions of TNFSF ligands are also differentially regulated during chronic kidney remodeling in oxalate-induced CKD.

### TNF superfamily ligands mRNA expressions in chronic immune complex glomerulonephritis

**Fig. 5:**
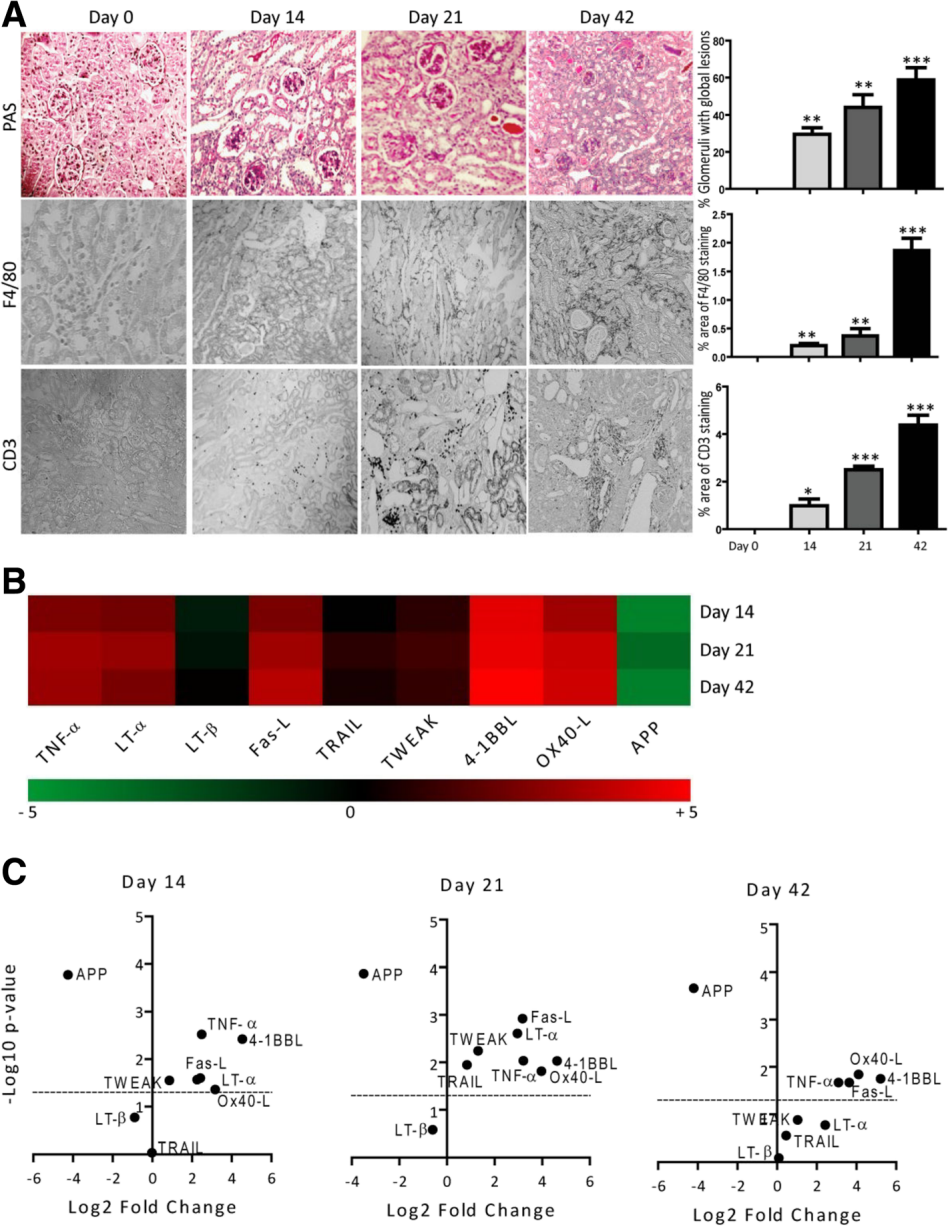
TNFSF ligands mRNA expressions in murine autologous anti-GBM nephritis. Autologous anti-GBM nephritis was induced in mice as described in methods. **a** Representative images of renal sections stained with PAS or for F4/80 and CD3. Original magnification: ×100. The glomerulosclerosis, F4/80, and CD3 positive cells were quantified. Data represents mean ± s.e.m. *p < 0.05, **p < 0.01, *p < 0.001. **b** Quantitative RTPCR was performed on cDNAs derived from the kidneys of mice on day 0, 14, 21 and 42. TNFSF ligands mRNA expression levels were normalised to 18 s rRNA expression levels. The heatmap represents the relative expression of TNFSF ligands mRNA levels at day 0, 14, 21 and 42 versus day 0. **c** The statistical analysis of TNFSF ligands mRNA expression levels in the kidneys at day 5, 10 and 35 versus control is represented using volcano graphs. *P* < 0.05 is considered statistically significant. Dotted line represent *p* = 0.05

TNFSF ligands play an important role in the pathogenesis of immune complex organ injuries [27]. Therefore, we studied the changes in their mRNA expression levels during an immune complex glomerulonephritis. We used an animal model of autologous anti-GBM nephritis and checked the expression of TNFSF ligands at day 14, 21, and 42 [28]. Immune complex glomerulonephritis was associated with a progressive increase in glomeruli with global lesions, as well as renal interstitial infiltration of F4/80+ macrophages and CD3+ T cells (Fig. 5a). Unlike, IRI and oxalate-induced CKD, the mRNA expression of LT-β remained low at all time points during the progression of immune complex glomerulonephritis (Fig. 5b-c). We observed a transient increase in the mRNA expression levels of TRAIL at day 21 (Fig. 5b-c). On the contrary, expression of 4-1BBL and TNF-α were significantly higher whereas APP expression was significantly lower at day 14, 21 and 42 (Fig. 5b-c). The mRNA expression of all other ligands was significantly higher during immune complex glomerulonephritis progression; however, TWEAK and LT-α induction was not statistically significant in the later phase at day 42 (Fig. 5b-c). Together, we observed sharp differences in kidney remodeling induced by persistent crystal injury and immune complex mediated injury.

## Discussion

The TNFSF ligands exert pleiotropic effects [11]. Although they evolved mainly for the regulation of immune cells in our body, over the years they have been reported to be involved in several pathogenic disorders as well. Here, our data demonstrate that alike PRRs [14], CLRs [16], TLR accessory molecules [15], and regulated necrosis-related molecules [18], the relative mRNA expression profiles of TNFSF ligands differ between physiological conditions in humans and mice, as well as between various pathophysiological conditions in mice.

All TNFSF ligands activate NF-ĸB signalling, albeit to different extent, therefore, the pathologies related to them mostly involve NF-ĸB signalling-mediated inflammation, for example – acute IRI. Several studies have reported the contribution of TNFSF ligands e.g. Fas-L, TWEAK and TNF-α, which are secreted by infiltrating immune cells, to intrarenal inflammation during IRI [29–32]. Accordingly, we observed a robust increase in the mRNA expression levels of TNFSF ligands after IRI that was also associated with increased intrarenal immune cell infiltration. Recently, TWEAK mRNA expressions were also reported to be increased in various mouse models of CKD such as lupus nephritis, immune complex glomerulonephritis, as well as rat and human diabetic nephropathy [32]. On the contrary, although TRAIL mRNA expression was increased at day 1 after IRI [27], we did not observe changes in the TRAIL mRNA expression at later time points in our study. Moreover, we observed that the expression levels of TNFSF ligands were highest during the healing phase after IRI, suggesting their putative involvement during tubule regeneration after an injury [33]. It may therefore interesting to explore whether TNFSF ligands contribute to regeneration/healing process upon acute kidney injury IRI.

Different functional states of immune cells regulate the process of regeneration upon kidney injury. Persistent inflammation is deleterious since it promote chronic tissue remodelling (fibrosis) [33]. We studied progressive kidney fibrosis using a murine model of oxalate-induced CKD. Although, we observed a robust increase in the mRNA expression levels of most TNFSF ligands in association with infiltrating immune cells, the expression patterns in progressive kidney injury were different from transient kidney injury observed in the IRI model. This suggests that different pathologies are driven by different TNFSF ligands. Interestingly, the injection of recombinant murine TNF-α in mice increased the expressions of TNF receptors (TNFRs) in the kidney [34]. Accordingly, the increased mRNA expression of TNF-α in kidneys of mice exposed to oxalate-rich diet can be linked to the already reported increased expression of TNFRs on tubular epithelium during the progression of oxalate-induced CKD [23]. TNFR induction supports the adhesion of calcium oxalate crystals to tubular epithelium and hence nephrocalcinosis eventually leading to CKD [23]. Moreover, TNF-α has also been reported to contribute to the pathogenesis of other forms of CKD – for example, diabetic nephropathy, Alport glomerulosclerosis, and chronic ischemic renal injury [35–37]. Therefore, it will be interesting to study the functional contribution of each of the other induced TNFSF ligands, as well as their receptors, during the progression of CKD.

In addition to the persistent trigger, chronic inflammation can also arise from tissue deposition of immune complex [38]. Surprisingly, unlike transient and persistent injuries, immune complex injury showed sharp differences in the mRNA expression patterns of TNFSF ligands, which did not at all correlate with the increased renal infiltration of immune cells. This suggests that the different pathomechanisms alter the mRNA expression of TNFSF ligands. Nevertheless, we observed a robust increase in the mRNA expression of TWEAK and TNF-α in the early phase, which was already shown to contribute to the disease pathology in mice, as well as human, kidney disease [32, 39–41]. Therefore, it would be interesting to study the specific contribution of all the induced genes in immune complex mediated renal injuries.

Furthermore, as TNFSF receptors mediate the effects of TNFSF ligands, the contribution of TNFSF ligands to disease pathologies also depend on TNFSF receptor expressions [3, 23, 42]. Hence, studying the organ- and species differences in expression of TNFSF receptors along with TNFSF ligands during health and disease conditions deserves careful consideration.

## Conclusions

We identified several TNFSF ligands that are induced during ischemic CKD, crystalline CKD, as well as immune complex glomerulonephritis. In addition, we have identified significant differences in the mRNA expression profiles of the TNFSF ligands in humans and murine solid organs during physiological conditions. Therefore, our data give robust reasons to consider these species-specific differences of TNFSF ligands in order to avoid the misinterpretation and wrong conclusions during data extrapolation between species. Since TNF-based therapeutics are already approved for human use, and several more are likely to be found in the future based on TNFSF ligands, the findings of our study recommend validating results of rodent studies, not only kidney diseases but also other pathogenic disorders, in human studies.
